# Supplementary material for: Assessing interface accuracy in macromolecular complexes
Source: PLoS One. 2025 Apr 2;20(4):e0319917. doi: 10.1371/journal.pone.0319917 (PMC11964455; doi:10.1371/journal.pone.0319917)
Supplement: S1 Table — (PDF) [file pone.0319917.s001.pdf]

S1 Table. Evaluation of the predicted 3D models from the RNA-protein docking decoys.

#	PDB ID	Residue mapping	I-INF	TM-score	DockQv2	DockQ Mapping	#Interactions		
							in target	in model	in both
**1**	2ANR	target: A:3-178;B:3-25<br>model: A:3-178;B:203-225	1.00	0.96	1.00	AB:AB	9	9	9
**2**	1HQ1	target: A:2-82;B:130-178<br>model: A:2-82;B:130-178	0.97	0.93	0.78	AB:AB	14	15	14
**3**	2ZUE	target: A:2-629;B:901-973<br>model: A:2-629;B:901-973	0.94	0.99	1.00	AB:AB	23	24	22
**4**	3OL9	target: A:1-461;B:596-612;C:688-701<br>model: M:1-461;N:596-612;O:688-701	0.93	0.98	0.99	ABC:MNO	21	22	20
**5**	2XDB	target: A:1-162;G:-3-32<br>model: A:1-162;G:-3-32	0.89	0.97	0.99	AG:AG	11	14	11
**6**	1J1U	target: A:1-306;B:501-574<br>model: A:1-306;B:501-574	0.88	0.87	0.97	AB:AB	7	9	7
**7**	3OVB	target: A:1-441;C:1-34<br>model: A:1-441;C:1-34	0.88	0.96	1.00	AC:AC	21	20	18
**8**	3FOZ	target: A:9-311;C:1-74<br>model: A:9-311;C:1-74	0.85	0.95	0.99	AC:AC	28	36	27
**9**	3EPH	target: A:13-421;E:2-71<br>model: A:13-421;E:2-71	0.84	0.98	1.00	AE:AE	38	38	32
**10**	1YVP	target: A:5-537;C:1-10;D:1-10<br>model: B:5-537;E:1-10;F:1-10	0.81	0.91	0.97	ACD:BEF	11	14	10
**11**	3LWR	target: A:11-337;B:3-55;C:4-123;D:1-58;E:5-17<br>model: A:11-337;B:3-55;C:4-123;D:1-58;E:5-17	0.79	0.92	1.00	ABCDE:ABCDE	29	43	28
**12**	2QUX	target: A:0-127;B:1-127;C:1-25<br>model: D:0-127;E:1-127;F:1-25	0.79	0.91	0.97	ABC:DEF	16	17	13
**13**	2ZM5	target: A:9-311;C:1-74<br>model: A:9-311;C:1-74	0.79	0.97	0.99	AC:AC	35	29	25

**14**	1QTQ	target: A:8-547;B:2-76<br>model: A:8-547;B:902-976	0.77	0.94	1.00	AB:AB	35	33	26
**15**	1R3E	target: A:1-308;C:404-420;D:404-420;E:404-420<br>model: A:10-317;C:404-420;D:404-420;E:404-420	0.76	0.97	0.18	ACDE:ACDE	28	27	21
**16**	1SJ3	target: C:6-97;B:100-172<br>model: P:6-97;R:100-172	0.72	0.94	0.99	CB:PR	13	18	11
**17**	1F7Y	target: O:2-87;B:1-57<br>model: A:1-86;B:1-57	0.71	0.95	0.43	OB:AB	17	17	12
**18**	1G1X	target: F:1-98;R:30-82;D:582-675;E:717-759<br>model: F:1-98;H:30-82;I:582-675;J:717-759	0.70	0.89	0.98	FRDE:FHIJ	11	9	7
**19**	1H4S	target: A:5-477;B:5-477;T:4-69<br>model: A:5-477;B:5-477;T:4-69	0.69	0.86	0.98	ABT:ABT	10	17	9
**20**	3MOJ	target: A:404-478;B:2508-2581<br>model: B:404-478;A:2508-2581	0.67	0.86	0.91	AB:BA	4	9	4
**21**	1GAX	target: A:1-862;C:901-975<br>model: B:1-862;D:901-975	0.66	0.93	1.00	AC:BD	21	25	15
**22**	2AKE	target: A:97-469;C:501-573<br>model: A:97-469;B:1-73	0.64	0.85	1.00	AC:AB	12	13	8
**23**	2GJW	target: A:62-305;B:62-305;E:3-20;F:3-14;H:15-21<br>model: A:65-308;B:64-307;E:3-20;F:3-14;H:15-21	0.61	0.85	0.41	ABEFH:ABEFH	18	12	9
**24**	1FFY	target: A:2-881;T:1-74<br>model: A:2-881;T:1-74	0.58	0.89	0.98	AT:AT	27	28	16
**25**	3DD2	target: H:16-245;B:1-25<br>model: H:16-245;B:1-25	0.57	0.94	0.99	HB:HB	6	13	5
**26**	2ZZM	target: A:2-334;B:1-72<br>model: A:2-334;B:1-72	0.56	0.77	0.93	AB:AB	25	33	16
**27**	1LNG	target: A:1-87;M:142-236<br>model: A:1-87;B:142-236	0.53	0.83	0.97	AM:AB	22	27	13
**28**	1N78	target: A:1-468;C:501-576<br>model: B:1-468;D:501-576	0.53	0.75	0.94	AC:BD	17	30	12

**29**	2BH2	target: A:62-305;B:62-305;E:3-20;F:3-14;H:15-21<br>model: A:65-308;B:64-307;E:3-20;F:3-14;H:15-21	0.52	0.76	0.94	AD:AC	19	28	12
**30**	2CSX	target: A:1-497;C:1-73<br>model: B:1-497;D:1-73	0.52	0.89	1.00	AC:BD	16	15	8
**31**	1KOG	target: A:242-642;B:242-642;I:69-105<br>model: C:242-642;D:242-642;K:69-105	0.51	0.88	0.99	ABI:CDK	8	17	6
**32**	2AZ0	target: A:2-71;B:2-72;C:1-18;D:1-18<br>model: A:2-71;B:2-72;C:1-18;D:1-18	0.50	0.84	0.86	ABCD:ABCD	16	16	8
**33**	1S03	target: H:3-129;B:1-47<br>model: H:3-129;A:1-47	0.49	0.79	0.96	HB:HA	6	11	4
**34**	1Q2R	target: A:11-382;E:25-44<br>model: C:11-382;F:25-44	0.48	0.83	0.99	CF:AE	14	20	8
**35**	1C0A	target: A:1-585;C:1-72<br>model: A:1-585;B:601-672	0.46	0.80	0.99	AC:AB	15	26	9
**36**	2RFK	target: A:11-337;C:1-21;D:1-25<br>model: A:11-337;D:1-21;E:1-25	0.44	0.76	0.98	ADE:ACD	14	18	7
**37**	1DFU	target: V:1-94;C:91-109;B:69-86<br>model: P:1-94;M:91-109;N:69-86	0.43	0.81	0.96	PMN:VCB	5	17	4
**38**	1F7U	target: A:5-607;B:901-973<br>model: A:5-607;B:901-973	0.42	0.70	0.93	AB:AB	24	29	11
**39**	3LLR	target: A:803-923;C:1-12;D:1-12<br>model: A:803-923;C:1-12;D:1-12	0.41	0.99	0.99	ACD:ACD	3	2	1
**40**	2NUG	target: A:3-218;B:3-220;C:2-12;D:2-12;E:17-27;F:17-27<br>model: A:3-218;B:3-220;C:2-12;D:2-12;E:17-27;F:17-27	0.41	0.76	0.81	ABCDEF:ABCDEF	24	57	15
**41**	1IL2	target: A:1-585;R:601-676<br>model: A:1-585;C:901-976	0.35	0.68	0.96	AR:AC	21	31	9
**42**	1JID	target: B:10-115;A:1-29<br>model: A:10-115;B:135-163	0.34	0.68	0.97	AB:BA	6	13	3
**43**	2ZKO	target: A:1-70;B:2-70;C:1-19;D:1-19<br>model: A:1-70;B:2-70;C:1-19;D:1-19	0.34	0.59	0.95	ABCD:ABCD	10	14	4

**44**	2ZNI	target: A:11-288;B:10-288;D:1-76<br>model: A:11-288;B:10-288;C:1-76	0.32	0.81	0.95	ABD:ABC	22	29	8
**45**	3FTF	target: A:7-246;C:1507-1528;D:1507-1528<br>model: A:7-246;C:1507-1528;D:1507-1528	0.32	0.69	0.94	ACD:ACD	8	5	2
**46**	3HHZ	target: A:2-422;R:1-45<br>model: O:2-422;R:1-45	0.31	0.81	0.96	AR:OR	7	6	2
**47**	1MMS	target: A:8-140;C:1051-1108<br>model: A:8-140;C:1051-1108	0.31	0.83	0.86	AC:AC	12	22	5
**48**	1R9F	target: A:25-148;E:1-19;F:1-19<br>model: A:25-148;B:1-19;C:1-19	0.27	0.59	1.00	AEF:ABC	10	12	3
**49**	3ADD	target: A:-1-248;C:1-76<br>model: A:-1-248;C:1-76	0.26	0.62	0.85	AC:AC	10	13	3
**50**	2HW8	target: A:5-228;B:2-38<br>model: A:5-228;B:2-37	0.26	0.45	0.67	AB:AB	23	16	5
**51**	1E80	target: A:5-75;B:2-95;E:100-148<br>model: C:5-75;D:2-95;E:100-148	0.26	0.91	0.94	ABE:CDE	4	15	2
**52**	2VPL	target: A:4-228;B:2-49<br>model: A:4-228;B:2-49	0.25	0.56	0.95	AB:AB	8	18	3
**53**	2UWM	target: A:441-633;E:13-35<br>model: A:441-633;C:13-35	0.25	0.61	0.67	AE:AC	15	10	3
**54**	1K8W	target: A:9-311;B:401-422<br>model: A:9-311;B:401-422	0.24	0.74	0.92	AB:AB	11	14	3
**55**	2DU3	target: A:1-534;C:901-971<br>model: A:1-534;D:901-971	0.24	0.93	1.00	AD:AC	3	6	1
**56**	3CIY	target: A:28-697;C:1-46;D:1-46<br>model: A:28-697;C:1-46;D:1-46	0.18	0.87	0.98	ACD:ACD	10	12	2
**57**	2FMT	target: B:1-314;A:1-76<br>model: A:1-314;C:1-76	0.17	0.49	0.97	BA:AC	7	20	2
**58**	2BTE	target: A:1-814;B:1-76<br>model: D:1-814;E:1-76	0.17	0.62	0.91	AB:DE	13	11	2

**59**	2R8S	target: A:4-226;B:1-212;C:103-260<br>model: H:1-223;L:1-212;R:103-260	0.11	0.43	0.29	HLR:ABC	17	21	2
**60**	2CZJ	target: A:3-122;B:1-72<br>model: E:4-123;F:1-72	0.11	0.84	0.50	AB:EF	9	10	1
**61**	1RC7	target: A:1-220;C:1-10;D:11-20;E:21-30;F:21-30<br>model: A:1-220;B:1-10;C:11-20;D:21-30;E:31-40	0.09	0.76	0.80	ACDEF:ABCDE	6	19	1
**62**	2IPY	target: A:2-889;D:1-30<br>model: A:2-889;C:1-30	0.07	0.36	0.66	CA:DA	37	22	2
**63**	1H3E	target: A:6-432;B:1-73<br>model: A:6-432;B:1-73	0.00	0.37	0.80	AB:AB	2	16	0
**64**	1JBS	target: A:1-149;C:1-29<br>model: A:1-149;C:1-29	0.00	0.86	0.98	AC:AC	0	7	0
**65**	1OOA	target: A:39-350;B:2-29<br>model: B:39-350;D:2-29	0.00	0.36	0.75	BD:AB	5	10	0
**66**	1SER	target: A:1-421;B:1-421;T:4-71<br>model: A:1-421;B:501-921;T:4-71	0.00	0.75	0.99	ABT:ABT	5	13	0
**67**	1T0K	target: Z:9-105;C:6-17;D:50-64<br>model: B:9-105;C:6-17;D:50-64	0.00	0.82	0.90	ZCD:BCD	3	6	0
**68**	1U0B	target: A:1-402;R:1-76<br>model: B:1-402;A:1-76	0.00	0.32	0.99	AR:BA	9	17	0
**69**	1UN6	target: A:104-190;F:4-115<br>model: C:104-190;E:4-115	0.00	0.64	0.72	AF:CE	7	8	0
**70**	2FK6	target: A:1-307;R:1-73<br>model: A:1-307;R:1-73	0.00	0.83	0.97	AR:AR	1	3	0
**71**	2V3C	target: B:2-427;A:142-236<br>model: C:2-427;M:142-236	0.00	0.37	0.75	BA:CM	23	19	0
**72**	3HL2	target: A:23-463;B:1-72<br>model: C:23-463;E:1-72	0.00	0.47	1.00	AB:CE	3	3	0
