# Supplementary figures and images for: Assessing interface accuracy in macromolecular complexes
Source: PLoS One. 2025 Apr 2;20(4):e0319917. doi: 10.1371/journal.pone.0319917 (PMC11964455; doi:10.1371/journal.pone.0319917)

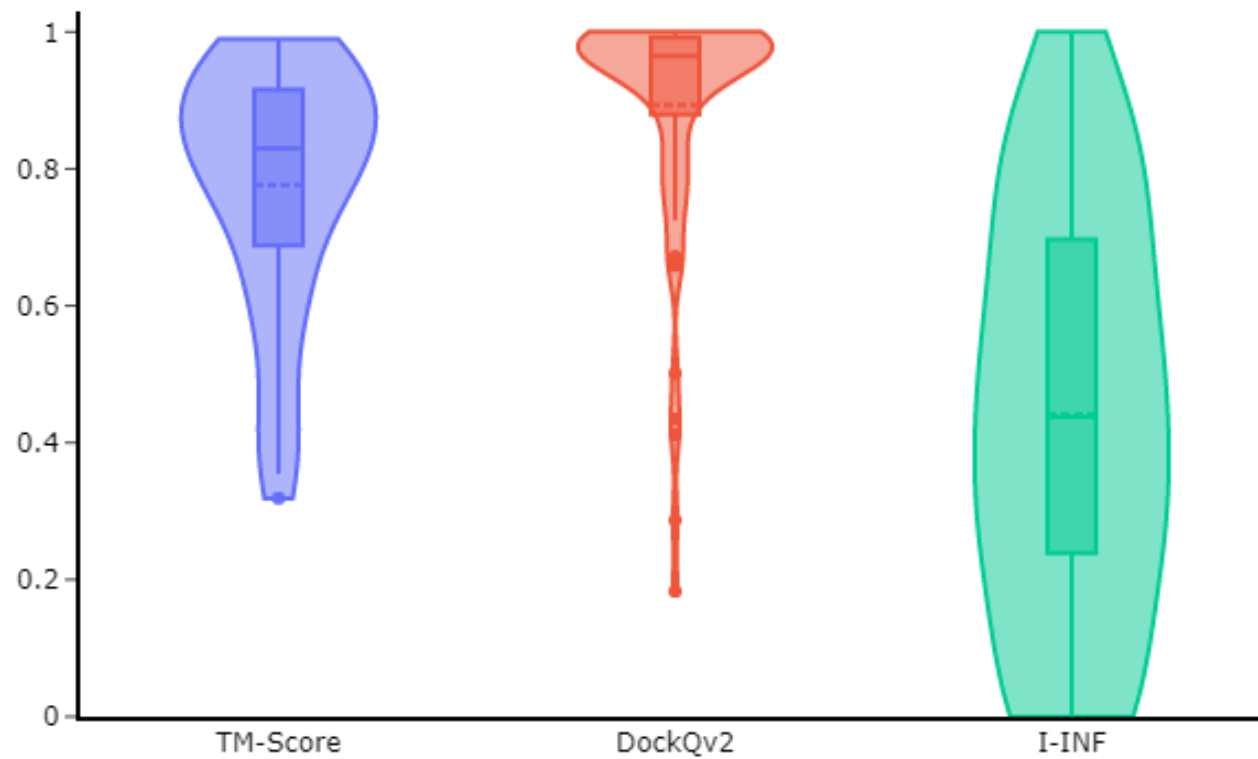

**S1 Fig. A distribution of TM-score, DockQv2, and I-INF values computed for the benchmark set.**

Supplement: S1 Fig — (PDF) [file pone.0319917.s002.pdf]
