# Supplementary material for: Assessing interface accuracy in macromolecular complexes
Source: PLoS One. 2025 Apr 2;20(4):e0319917. doi: 10.1371/journal.pone.0319917 (PMC11964455; doi:10.1371/journal.pone.0319917)
Supplement: Attachment — Submitted filename: response-to-referee.pdf [file pone.0319917.s004.pdf]

We thank the Reviewer for the supplementary suggestion. We have answered it in the revised version of the manuscript, and below we provide detailed explanations:

Reviewer's comment: I previously commented that 'TM-score and I-INF do NOT measure the same aspect of complex assembly. I-INF only evaluates the correctness of the interface, while TM-score evaluates the global topology. There should be some theoretical justification on why TM-score has a high correlation with I-INF.' This revision only answered my question in the response letter, but did not make any corresponding modification in the manuscript.

Response: Indeed, in the previous version of the manuscript, we did not include the appropriate modification in response to this comment. We have now addressed this. In the "Results and Discussion" section, we have inserted the following text:

“Although TM-score evaluates the global topology of the model and I-INF specifically assesses the accuracy of the intermolecular interface, we observed a high Pearson correlation (0.73) between these metrics. This suggests that, in our dataset, a correctly predicted global fold is largely a consequence of the proper spatial arrangement of the molecular components, resulting in accurately modeled interfaces. In other words, deviations in the overall fold are primarily associated with errors in the interface regions. Therefore, high TM-score values frequently coincide with high I-INF values, highlighting our models' interdependence between global structural accuracy and interface correctness.”

We hope that this addition provides the necessary theoretical justification for the observed high correlation between TM-score and I-INF.
